# Single-step manufacturing of hierarchical dielectric metalens in the visible

**DOI:** 10.1038/s41467-020-16136-5

**Published:** 2020-05-08

**Authors:** Gwanho Yoon, Kwan Kim, Daihong Huh, Heon Lee, Junsuk Rho

**Affiliations:** 1 Department of Mechanical Engineering, Pohang University of Science and Technology (POSTECH), Pohang, 37673 Republic of Korea; 2 Department of Materials Science and Engineering, Korea University, Seoul, 02841 Republic of Korea; 3 Department of Chemical Engineering, Pohang University of Science and Technology (POSTECH), Pohang, 37673 Republic of Korea; 4 National Institute of Nanomaterials Technology (NINT), Pohang, 37673 Republic of Korea

**Keywords:** Nanophotonics and plasmonics, Metamaterials, Design, synthesis and processing, Surface patterning

## Abstract

Metalenses have shown a number of promising functionalities that are comparable with conventional refractive lenses. However, current metalenses are still far from commercialization due to the formidable fabrication costs. Here, we demonstrate a low-cost dielectric metalens that works in the visible spectrum. The material of the metalens consists of a matrix-inclusion composite in which a hierarchy satisfies two requirements for the single-step fabrication; a high refractive index and a pattern-transfer capability. We use a UV-curable resin as a matrix to enable direct pattern replication by the composite, and titanium dioxide nanoparticles as inclusions to increase the refractive index of the composite. Therefore, such a dielectric metalens can be fabricated with a single step of UV nanoimprint lithography. An experimental demonstration of the nanoparticle composite-based metalens validates the feasibility of our approach and capability for future applications. Our method allows rapid replication of metalenses repeatedly and thereby provides an advance toward the use of metalenses on a commercial scale.

## Abstract (web summary)

Current metalenses are far from commercialization due to fabrication cost and low throughput. Here, the authors use a UV-curable resin as a matrix for direct pattern replication by the composite and TiO2 nanoparticles to increase the refractive index of the composite, allowing dielectric metalenses to be manufactured in a single step.

## Introduction

Metasurfaces that consist of ultrathin subwavelength antenna arrays drive a paradigm shift from bulky and heavy to compact and light optics^1,2^. The strong interaction between electromagnetic (EM) waves and antenna allows efficient light modulation at the subwavelength scale^3^, in contrast to typical refractive components that operate at the wavelength scale. Metasurfaces can control the amplitude, phase, polarization, and frequency of EM waves by adjusting the configuration of antennas, and can realize several promising applications, such as diffraction-limited imaging^4,5^, high-resolution holography^6–8^, optical cloaking^9^, and structural color printing^10,11^. Simultaneous control of more than one wave property is also feasible with a single antenna enabling multifunctional operations^12,13^. Furthermore, multilayered metasurfaces provide a great opportunity for functional integration^14,15^.

Metalenses designed to focus light have emerged as next-generation lenses. Spherical aberration can be perfectly removed in metalenses without trouble whereas conventional aspherical lenses require costly and time-consuming processes^16^. Early plasmonic metalenses suffered from low diffraction efficiencies^17^ that were drastically increased by using dielectric antennas that induce strong electric and magnetic resonances^3^. Geometric phase from anisotropic antennas realizes broadband operation with polarization dependence^18,19^, and is favorable for use in augmented reality devices^20^. Propagation phase from isotropic antennas enables the polarization-insensitive operation of metalenses^21^. Appropriate control of both the geometric and propagation phase of antennas can compensate for chromatic aberration of metalenses which is a characteristic of diffractive optical elements^22,23^. The chromatic aberration can also be eliminated by other methods such as transmission amplitude modulation^14^ or algorithm-assisted optimization^24^. Dielectric metalenses have the potential to replace conventional bulky and heavy lenses, but fabrication limitations impede the commercialization of metalenses.

The fabrication of dielectric metalenses suffers from both high manufacturing costs and low throughput. Electron beam lithography (EBL) is widely used to fabricate metalenses because they require subwavelength structures of sizes smaller than the diffraction limit of photolithography. The wavelength of an electron beam is only a few nanometers, so EBL can define metalenses with high pattern fidelity but the extremely low throughput hinders large-scale fabrication^25^. Nanoimprint lithography (NIL) has been evaluated as a way to manufacture metasurfaces on large scale^26,27^. The fabrication of the master molds for NIL requires EBL, but the replication from the master mold is much faster than EBL. However, conventional NIL also requires secondary operations such as thin-film deposition and etching^20^, so the production of metalenses by NIL is not yet competitive with the production of commercial plastic lenses using injection molding^28^. These secondary operations reduce the productivity and degrade the substrate compatibility of NIL. Hence, the commercialization of metalenses requires the development of a one-step fabrication method.

Here, a low-cost hierarchical dielectric metalens is demonstrated using a single step of NIL. The fundamental requirement is that the pattern transferred from the mold should itself work as a metalens. Metalens patterns can be transferred to ultraviolet (UV)-curable resin by NIL, but the refractive index *n* of typical resin is too low to work as a meta-atom^29^. Some commercial hybrid inorganic–organic materials have high *n* in the visible range, but they suffer from high shrinkage and limited film thickness^30^. Therefore, we develop a nanoparticle composite (NPC) consisting of dielectric-NP inclusion in a matrix of UV-curable resin, and such a hierarchy enables the realization of low-cost dielectric metalenses. Titanium dioxide (TiO_2_) NPs are used in this work. TiO_2_ has much higher *n* than the matrix, so the effective *n* of the NPC can be increased and controlled by the content of TiO_2_ NPs. The matrix of the NPC is hardened by UV illumination, so the NPC pattern can be directly transferred from the mold and work as a metalens without any secondary operations. The final metalens is designated for a plum pudding metalens (PPML) because its unit structure resembles a plum pudding. Experimental demonstration of the PPML verifies the feasibility of our approach yielding a focusing efficiency of 33% at a design wavelength *λ*_d_ = 532 nm, and the efficiency can be increased by further optimization of the structure configuration. The maximum aspect ratio of the final NPC structures can exceed 10:1 due to the high stiffness of the NPC after UV curing. Large-scale fabrication is also feasible using large-sized master molds. Since intermediate soft molds are reusable, the PPML can be replicated repeatedly using only a single step of printing without any secondary operations. The absence of secondary operations allows the fabrication of the PPML on flexible substrates which are usually vulnerable to plasma damage. Therefore, our approach will provide real opportunities for practical applications of metalenses.

## Results

### Characteristics of TiO_2_ NPC

**Fig. 1:**
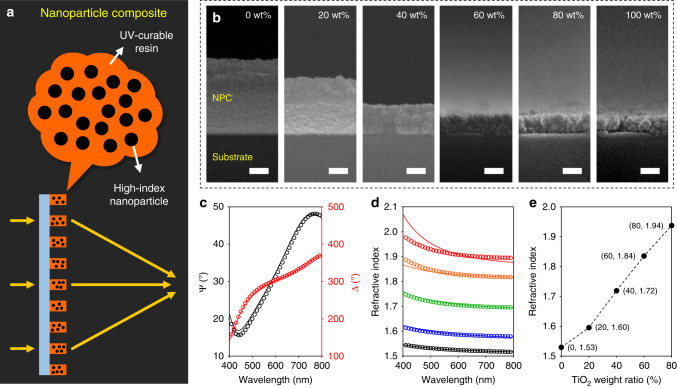
Characterization of the nanoparticle composite. **a** Schematic of the plum pudding metalens (PPML). Yellow lines: light propagation direction through the PPML. The PPML can be fabricated with a single step of printing at low cost due to the compatibility of the nanoparticle composite (NPC). The refractive index of the NPC is high enough to realize metalenses, so the NPC can be directly used as a material of metalenses without any secondary operations such as thin-film deposition or etching. **b** Scanning electron micrographs of cross sections of the spin-coated NPC films with different weight ratios of titanium dioxide (TiO_2_) nanoparticles. Spin-coating conditions are the same for all samples. The particle size is much smaller than the operating wavelength, so the NPC can work as a homogeneous effective medium. All scale bars: 150 nm. **c** Ellipsometry analysis of the NPC film with 80% of the TiO_2_ weight ratio. The amplitude component is denoted by *Ψ* while the phase difference is represented as *Δ*. Real lines: theoretical prediction by the Cauchy dispersion model; circles: experimental measurements. **d** Comparison of the refractive index of the NPC films. Real lines: retrieved data from the ellipsometry measurement of each NPC film with different TiO_2_ weight ratio (black: 0%; blue: 20%; green: 40%; orange: 60%; red: 80%); circles: calculated data by the Maxwell–Garnett formula with different volume fraction of TiO_2_ nanoparticles (Black: 0%; blue: 7%; green: 20%; orange: 33%; red: 41%). **e** The measured refractive index variation of the NPC films by the weight ratio at the wavelength of 532 nm. The dashed line is to guide the eye.

The PPML has a hierarchy of NPs embedded in a UV-curable resin (Fig. 1a). The material of the PPML must satisfy two requirements to realize low-cost metalens fabrication: a sufficiently-high *n* to work as a meta-atom, and feasible transfer of the mold pattern. Therefore, we use the NPC as a material of the PPML to achieve those requirements. The NPC is a matrix-inclusion composite. The UV-curable resin is used as a matrix material to enable the replication of the mold pattern, and TiO_2_ NPs are embedded in the inclusions to increase the effective *n* of the NPC. For the NPC to be a homogeneous effective medium, the TiO_2_ NPs should be much smaller than the operating wavelength^31^, so 30 nm NPs are used in this work.

The *n* of the NPC is characterized both theoretically and experimentally. The *n* of the NPC is closely related to the filling fraction of the TiO_2_ NPs, so we characterize the optical properties of the NPC film by changing the ratio of TiO_2_ NPs. We prepare six kinds of NPCs from 0% to 100% of the TiO_2_ weight ratio (see Method), and they are spin-coated on a silicon substrate with the same spin-coating conditions (Fig. 1b). After UV curing, the final thickness of the NPC film decreases as the weight ratio increases due to the reduction in the amount of monomer. The coated NPC films are analyzed using ellipsometry, and the measured data is fitted using the Cauchy dispersion model which is expressed as

1 $$n\left( \lambda \right) = A + \frac{B}{{\lambda ^2}} + \frac{C}{{\lambda ^4}},$$

where *n* is the refractive index, *λ* is the wavelength, and *A*, *B*, *C* are coefficients to match the model with the measured data. For example, the NPC film with 80% of the TiO_2_ weight ratio can be described by the model with coefficients of *A* = 1.853, *B* = 0.00864, and *C* = 0.00414 (Fig. 1c). The resulting mean square error between the model and the data is 23.7 which is low enough to validate that indeed the NPC acts as a homogeneous effective medium in the visible regime. The *n* of the model is proportional to the TiO_2_ weight ratio, so the *n* of the NPC can be controlled over a broad range by adjusting the weight ratio (Fig. 1d).

We also calculate the *n* of the NPC by using the Maxwell–Garnett formula of the effective medium theory which yields the effective permittivity of the medium as^32^

2 $$\varepsilon _{{\mathrm{eff}}} = \varepsilon _{\mathrm{m}} + \beta \left( {\varepsilon _i - \varepsilon _{\mathrm{m}}} \right)\frac{{3\varepsilon _{\mathrm{m}}}}{{\varepsilon _i + 2\varepsilon _{\mathrm{m}} - \beta \left( {\varepsilon _i - \varepsilon _{\mathrm{m}}} \right)}},$$

where *ε*_m_ and *ε*_*i*_ represent complex permittivity of the matrix and inclusion material respectively, and *β* is the volume fraction of inclusions. The *n* can be derived from the permittivity (Eq. 2), and the measured data agrees with the calculation results enabling deduction of the volume fraction of TiO_2_ NP in each NPC film. Some deviations result from the formula assuming perfectly-uniform sphere-shaped NP whereas the actual NPC films contain non-uniform random-shaped NP. Since the NPC with 100% of the TiO_2_ weight ratio cannot be used for patterning due to the absence of monomer and photo-initiator, the maximum *n* of 1.94 at the wavelength of 532 nm can be achieved near the weight ratio of 80% (Fig. 1e), and the corresponding volume fraction of TiO_2_ NPs is approximately 40%. This is the condition used in this work.

### Design and simulation of metalens

**Fig. 2:**
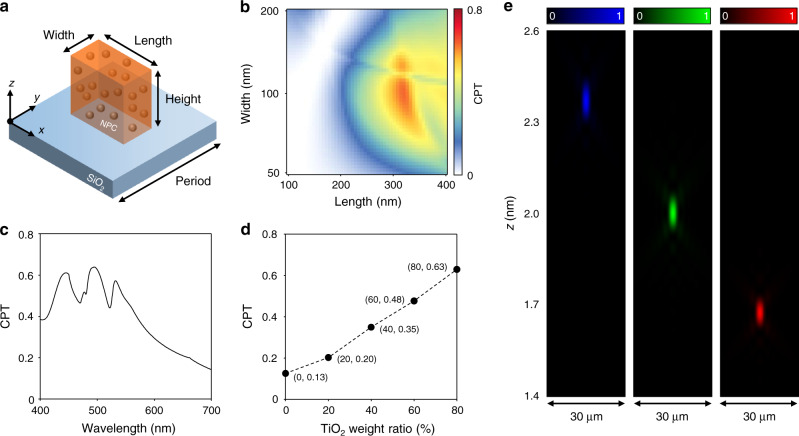
Design and simulation of plum pudding metalens. **a** Configuration of unit structure consists of nanoparticle composite (NPC). Length: 330 nm; width: 100 nm; height: 720 nm; period: 450 nm. Spheres inside the structure represent titanium dioxide (TiO_2_) nanoparticles. **b** Calculation results of cross-polarization transmittance (CPT) of the NPC-based nanorod at the wavelength *λ* = 532 nm. Height: 720 nm; period: 450 nm. **c** The calculated CPT of the unit structure. **d** The maximum CPT variation by the TiO_2_ weight ratio at *λ* = 532 nm when the height and period are fixed as 720 and 450 nm, respectively. The dashed line is to guide the eye. **e** Optical field simulation of the metalens based on the Rayleigh–Sommerfeld diffraction formula with (left) *λ* = 450 nm, (center) *λ* = 532 nm, (right) *λ* = 635 nm. The metalens is located at *z* = 0, and designed to have *f*_d_ = 2 mm at *λ*_d_ = 532 nm. The light passes through the metalens, then propagates along +*z* direction. Each color represents a normalized field intensity in the *xz* plane. Due to chromatic aberration, the designed phase gradient is not valid for other wavelengths resulting different focal lengths of 2.36 mm for *λ* = 450 nm and 1.67 mm for *λ* = 635 nm.

Rigorous coupled-wave analysis (RCWA) is used to simulate the optical properties of the NPC-based nanostructures with the measured *n*. Anisotropic rectangular nanorods induce geometric phase of the transmitted cross-polarized light by structure rotation. Broadband operation is a unique advantage of geometric phase due to its wavelength independence, so we use rectangular nanorods as the unit structure of the PPML (Fig. 2a). Cross-polarized light is affected by geometric phase whereas transmission of co-polarized light is irrelevant to structure rotation. Therefore, cross-polarization transmittance (CPT) is directly connected to the focusing efficiency of the PPML. When a left-handed circularly polarized (LCP) beam is normally incident to the unit structure, the Jones vector of the outgoing wave can be written as^33^

3 $$\frac{{T_{\mathrm{L}} + T_{\mathrm{S}}}}{2}\left[ {\begin{array}{*{20}{c}} 1 \\ i \end{array}} \right] + \frac{{T_{\mathrm{L}} - T_{\mathrm{S}}}}{2}{\mathrm{exp}}(i2\alpha )\left[ {\begin{array}{*{20}{c}} 1 \\ { - i} \end{array}} \right],$$

where *T*_L_ and *T*_S_ represent the complex transmission coefficients under light incidence of linear polarization along the long and short axis of the nanorod respectively, and *α* is the rotation angle of the nanorod along the *z-*axis. The CPT is the magnitude of the cross-polarization component (Eq. 3), and we calculate the CPT of the nanorod by changing its length from 100 to 400 nm and width from 50 to 200 nm (Fig. 2b). These ranges are determined considering fabrication compatibility. The designed unit structure yields higher CPT at short wavelengths in the visible; this high CPT is a result of the near-zero extinction coefficient of the NPC (Fig. 2c).

The higher *n* of the NPC usually benefits from the higher CPT. We calculate the CPT of rectangular nanostructures based on the measured *n* of the NPC. The whole relation between the CPT and *n* is not simple at all, so we fix the structure height as 720 nm in the calculation to investigate local effects of the *n* near the structural condition used in this work (Supplementary Fig. 1). As a result, we verify that the maximum CPT in the range of length and width is proportional to the weight ratio of TiO_2_ NPs (Fig. 2d).

The PPML is designed to follow a quadratic phase gradient to focus transmitted cross-polarized light without spherical aberration. The required phase gradient for the PPML is described as^4^

4 $$\varphi \left( {x,y} \right) = \frac{{2\pi }}{{\lambda _{\mathrm{d}}}}\left( {f_{\mathrm{d}} - \sqrt {x^2 + y^2 + f_{\mathrm{d}}^2} } \right),$$

where *λ*_d_ = 532 nm is the design wavelength, *f*_d_ = 2 mm is the design focal length, *x* and *y* are coordinates of each nanorod. Each nanorod in the PPML requires *α* = *φ*/2 at each position (Eq. 3). The focusing characteristic can be simulated using the Rayleigh–Sommerfeld diffraction formula^34^ by discretizing the phase gradient by the sampling period *P* = 450 nm and the diameter *D* = 450 μm of the PPML (Fig. 2e). At *λ* = *λ*_d_, transmitted cross-polarized light is focused at *f* = *f*_d_ as designed. Although the quadratic phase gradient is not valid for two other wavelengths of *λ* = 450 and 635 nm, they can be still focused, but at different positions resulting from chromatic aberration. The simulated focal lengths are 2.36 mm for *λ* = 450 nm and 1.67 mm for *λ* = 635 nm. As *λ* increases, the focal length of diffractive lenses decreases whereas that of a refractive lens increases^35^.

### Fabrication of PPML

**Fig. 3:**
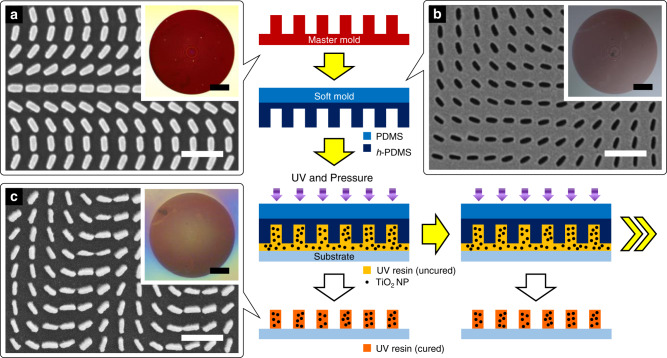
Fabrication schematic of plum pudding metalens. A master mold is fabricated by electron beam lithography and coated by the self-assembled monolayer (SAM) to improve mold release. Hard polydimethylsiloxane (*h*-PDMS) is coated on the master mold, then a polydimethylsiloxane (PDMS) buffer layer is spread on the *h*-PDMS layer. A released soft-mold is also coated by the SAM to facilitate mold release in succeeding processes. Nanoparticle composite (NPC) which consists of titanium dioxide (TiO_2_) nanoparticles (NPs) in a matrix of UV-curable resin is dropped on a glass substrate, and then it is covered by the soft-mold. UV light illumination with appropriate pressure hardens the NPC in the form of the master mold. The replication process is completed by releasing the reusable soft-mold from the substrate, and the soft-mold is reusable for additional replication. The final NPC patterns have a residual layer, but it is negligible because structures are much higher than the residual layer. **a**–**c** Scanning electron micrographs of the master mold, soft mold, and final plum pudding metalens, respectively. All scale bars: 1 μm. (insets) Optical micrographs of each. All scale bars: 100 μm.

The PPML is replicated from the master mold using a single step of UV-NIL. EBL is used to fabricate the master mold^36^ (Fig. 3a), and it is coated by a self-assembled monolayer (SAM) that reduces the surface energy to facilitate mold release^37^. Hard polydimethylsiloxane (*h*-PDMS) is prepared by a typical method of mixing a vinyl polydimethylsiloxane (PDMS) prepolymer, modulator, hydrosilane prepolymer, platinum catalyst, and toluene^38^. Then, a stiff layer of *h*-PDMS is coated on the master mold covering the entire structure, and a flexible buffer layer of PDMS is coated on the *h*-PDMS layer (Fig. 3b). This composite soft-mold allows high-resolution pattern transfer due to the low viscosity and high stiffness of *h*-PDMS, which makes a soft-mold reusable. The compression modulus of the *h*-PDMS can widely vary from 0 to 10 N mm^−2^ depending on the vinyl ratio in prepolymer and hydrosilane^39^. The minimum feature size of the *h*-PDMS with the compression modulus of 9.7 N mm^−2^ is as small as 80 nm, whereas pure PDMS in which modulus is 2 N mm^−2^ is usually capable of patterning down to 400 nm-sized structures. The SAM is also coated on the released soft-mold to facilitate mold release in succeeding processes^40^. The NPC is prepared by blending methyl isobutyl ketone (MIBK) dispersed by 30-nm TiO_2_ NPs with a mixture of a monomer, photo-initiator and MIBK^41^. Two solutions use the same solvent, so they can be mixed without any solute precipitation. The NPC is dropped on the glass substrate, then covered with the soft-mold. UV illumination with appropriate pressure hardens the NPC in the form of the master mold. After the release of the soft-mold, the PPML is created on the substrate (Fig. 3c).

**Fig. 4:**
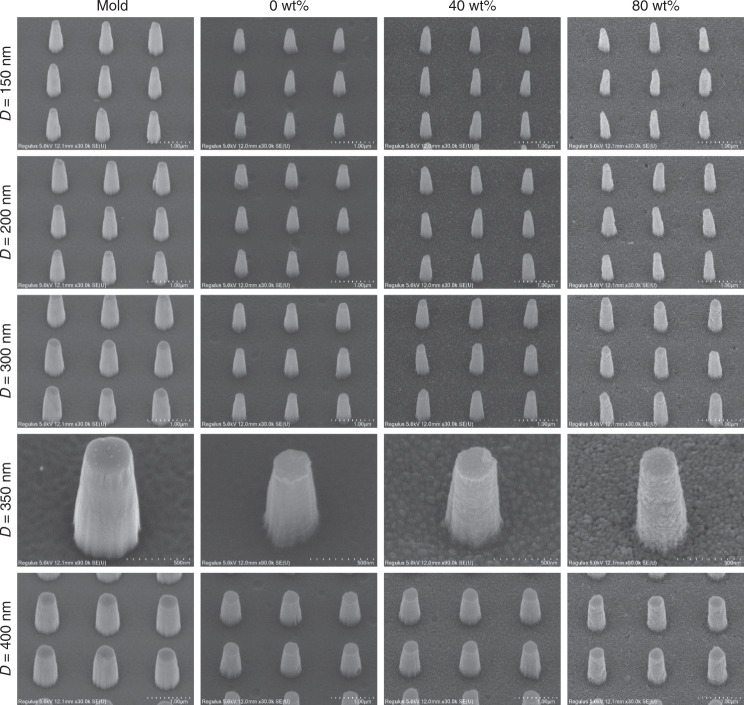
Comparison of fabrication quality for circular nanopillar arrays. The titanium dioxide weight ratio varies from 0 to 80% while the diameter *D* of circular nanopillars changes from 150 to 400 nm. The height of nanopillars of the master mold is 1.2 µm, and all the patterns are tilted by 30°. It is worth noting that the electron beam of a scanning electron microscope deforms the nanoparticle composite patterns, i.e., bending and height shrinkage.

**Fig. 5:**
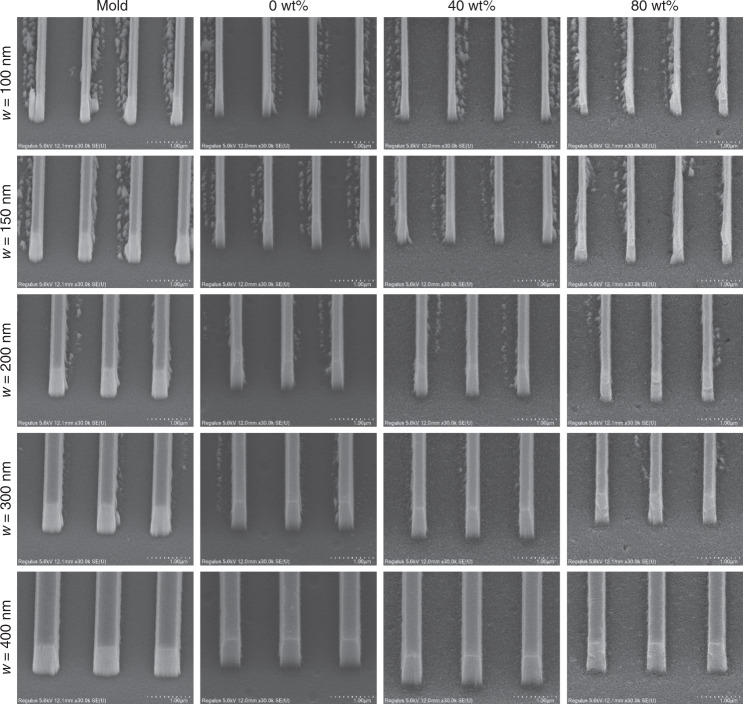
Comparison of fabrication quality for line arrays. The titanium dioxide weight ratio varies from 0 to 80% while the width *w* of lines changes from 100 to 400 nm. The height of line structures of the master mold is 1.2 µm, and all the patterns are tilted by 30°. It is worth noting that the electron beam of a scanning electron microscope deforms the nanoparticle composite patterns, i.e., bending and height shrinkage.

Our fabrication method features rapid and high-resolution patterning of high-aspect-ratio dielectric nanostructures by a single process with strong substrate compatibility. Once the master mold is prepared, a single step of UV-NIL is enough to fabricate the PPML on the substrate without any secondary operations. UV-NIL is a rapid process, and the soft-mold is reusable; therefore, the PPML is highly competitive to commercial plastic lenses by injection molding. Due to the high stiffness of the NPC after UV curing, the aspect ratio of transferred patterns exceeds 10:1 which is not easy to achieve using typical sol–gel approaches^42^. The final NPC patterns have 20% shrinkage along each dimension regardless of the filling fraction of TiO_2_ NPs (Fig. 4), and the minimum linewidth of NPC patterns is approximately 100 nm (Fig. 5). The absence of secondary operations such as etching and annealing allows fabrication of the PPML on flexible substrates that are usually vulnerable to plasma and heat. Curved substrates are also compatible with our method due to the flexibility of the soft-mold. Therefore, our method is highly favorable for the fabrication of dielectric metasurfaces compared with conventional NIL (Supplementary Table 1).

### Optical characterization of metalens

**Fig. 6:**
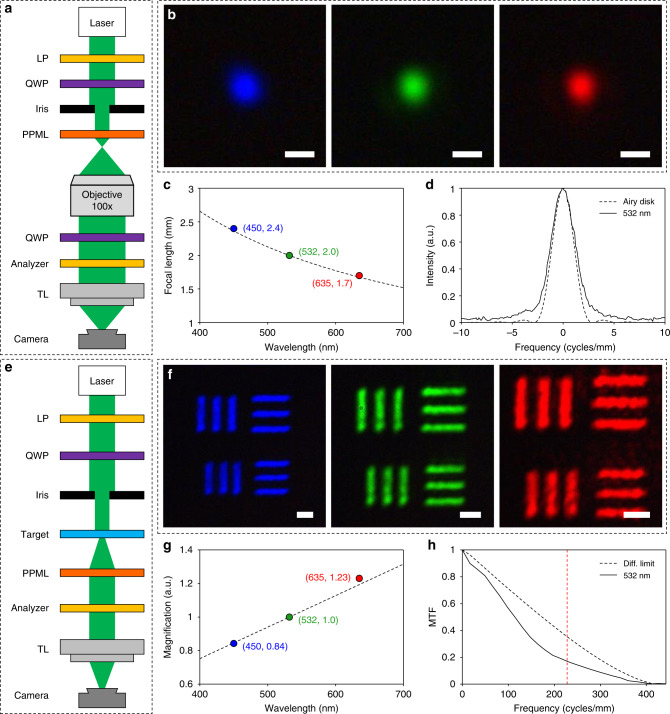
Optical properties of plum pudding metalens. **a** Optical setup to characterize the focal spot. LP linear polarizer, QWP quarter-wave plate, TL tube lens, PPML plum pudding metalens. **b** Captured images of focal spots at wavelengths *λ* = 450, 532, and 635, respectively. All scale bars: 5 μm. **c** Measured focal lengths denoted by three colored circles of 2.4 mm at *λ* = 450 nm, 2.0 mm at *λ* = 532 nm and 1.7 mm at *λ* = 635 nm. Dashed line: theoretical prediction of the focal length *f* = *f*_d_*λ*_d_/*λ* where *λ*_d_ and *f*_d_ represent the design wavelength (532 nm) and focal length (2 mm), respectively. **d** Comparison of focal spot intensity profiles at *λ* = 532 nm. The full width at half maximum (FWHM) of the measured profile is 2.8 μm that is closely consistent with the FWHM of the airy disk of 2.4 μm. **e** Optical setup to evaluate imaging properties of the PPML by using it as an objective lens. The analyzer allows only cross-polarized light to reach the camera. **f** Captured target images at *λ* = 450, 532, and 635 nm, respectively. The imaging area includes the element 5 and 6 of group 7 in the negative 1951 United States Air Force resolution test chart. All scale bars: 5 μm. **g** Comparison of magnification by wavelengths. Dashed line: theoretical prediction of the relative magnification. Colored dots: measured image magnification at each wavelength. **h** Modulation transfer function (MTF) comparison between the PPML and a diffraction-limited imaging system. Red dashed line: location of the frequency value of 228 mm^−1^ that is the maximum spatial frequency of the target image.

The focusing properties of the PPML are characterized using a customized optical setup (Fig. 6a). An LCP beam is normally incident to the PPML from the substrate to the structures. The iris tailors the incident beam to match the size of the PPML. The diverging beam behind the focal point is collected by a commercial objective lens of numerical aperture (NA) 0.7, then passes through an analyzer that filters out the co-polarization component. Therefore, the camera receives only cross-polarized light that has right-handed circularly polarization (RCP) corresponding to a point spread function (PSF) of the PPML. The PSF for each wavelength is measured at each focal point (Fig. 6b). The focusing efficiency *η* is defined as the ratio of optical power of the focal spot to the total incidence. Only the cross-polarized beam is focused at the focal point, so *η* can be considered to be the CPT of unit structures. The measured *η* of the PPML are 45% at *λ* = 450 nm, 33% at *λ* = 532 nm and 11% at *λ* = 635 nm. Due to fabrication defects, these are less than the calculated CPT, but the trends are similar.

Focal spots at each wavelength have the same full width at half maximum (FWHM) due to the low NA of the PPML. The measured focal lengths are 2.4 mm at *λ* = 450 nm, 2.0 mm at *λ* = 532 nm and 1.7 mm at *λ* = 635 nm, which agree well with the theory of the focal length of diffractive lenses *f* ≈ *f*_d_*λ*_d_/*λ* where *λ* represents a working wavelength^35^ (Fig. 6c). Considering *D* = 450 μm, NAs of the PPML can be calculated as 0.09 at *λ* = 450 nm, 0.11 at *λ* = 532 nm and 0.13 at *λ* = 635 nm by using the measured focal length for each wavelength. The measured FWHM of focal spots is 2.8 μm, which corresponds to a diffraction-limited FWHM^4^ of *λ*/(2 × NA) = 2.4 μm that is consistent for each wavelength due to the low NA (Fig. 6d). The diffraction-limited FWHM can also be derived from the Airy disk that represents the PSF formed by a perfect lens. The intensity of the airy disk is described as^43^

5 $$I = I_0\left( {\frac{{2{\cal{J}}_1\left( {\frac{{\pi Dr}}{{\lambda f}}} \right)}}{{\frac{{\pi Dr}}{{\lambda f}}}}} \right)^2,$$

where *I*_0_ is the maximum intensity, *r* is the radial distance from the center, and *J*_1_ is the Bessel function of the first kind of order one.

The imaging characteristics of the PPML are characterized by using it as an objective lens (Fig. 6e). An LCP beam is normally incident to the target of the negative 1951 United States Air Force resolution test chart, and the transmitted beam from the target is collected by the PPML. This beam also passes through the analyzer to filter out the co-polarization component, so only the RCP beam reaches the camera. The imaging area includes elements 5 and 6 of group 7 of the target; the field of view can be further enlarged by increasing the PPML size (Fig. 6f). The captured images show the same object with different magnifications by each wavelength due to the chromatic aberration of the PPML. Measured magnifications are proportional to the working wavelength and are therefore 0.84 at *λ* = 450 nm and 1.23 at *λ* = 635 nm, which are normalized to the magnification at *λ* = 532 nm (Fig. 6g). The positions of optical components behind the PPML are fixed, so the magnification is inversely proportional to the focal length of the PPML. The focal length is also inversely proportional to the working wavelength; therefore, the magnification is proportional to the working wavelength.

A modulation transfer function (MTF) provides information on the resolution and contrast that can be achieved by an optical imaging system. The MTF is defined as the magnitude of the Fourier transform of the square of an impulse response of the optical system normalized by a zero-frequency value. The impulse response of the optical imaging system corresponds to the PSF, so the MTF of our PPML-based imaging system can be described as^43^

6 $${\mathrm{MTF}} \equiv \left| {\frac{{{\int} {{\int} {I\left( {x,y} \right){\mathrm{exp}}\left[ { - i2\pi \left( {f_{\mathrm{x}}x + f_{\mathrm{y}}y} \right)} \right]dxdy} } }}{{{\int} {{\int} {I\left( {x,y} \right)dxdy} } }}} \right|,$$

where *I*(*x*,*y*) denotes the PSF, *f*_x_ and *f*_y_ represent spatial frequencies along *x*-axis and *y*-axis, respectively. The cut-off frequency of our system reaches 400 mm^−1^ (Fig. 6h). This is much higher than the spatial frequency of the imaging target of 228 mm^−1^, so our system can distinguish each line pair of the target. The MTF of a diffraction-limited imaging system can be calculated by using the airy disk as the PSF due to the physical meaning of the Airy disk (Eq. 5). The FWHM of the PSF by the PPML is analogous to the airy disk. Therefore, the cut-off frequency of the diffraction-limited system is also analogous to our system whereas noise in the PSF by the PPML causes contrast reduction in our imaging system below the cut-off frequency compared with the diffraction-limited system.

## Discussion

The experimental demonstration verifies the feasibility of the PPML as a practical low-cost metalens, and the current PPML can be further improved for future applications. The optical property of the NPC has a critical function in the PPML because the thickness of unit structures can be reduced by increasing the *n* of the NPC. Our fabrication method can transfer structures that have high-aspect ratios, but reduction in structure thickness increases the reliability of the fabrication of the PPML. Mechanical stability of the current NPC can be further increased by using a hard material as the matrix. The material should be viscous to replicate the mold pattern but become mechanically stable after solidification by UV illumination or annealing. If the height of NPC structures reaches 1 µm without any structural defects such as bending, the focusing efficiency of the PPML can exceed 85% at *λ* = 532 nm (Supplementary Fig. 2). Although chromatic aberration is not corrected in this work, our method is also capable of realizing achromatic metalenses by stacking individual metalenses and phase correctors. It has been already reported that stacked metasurfaces including metalenses can improve imaging qualities in various ways such as coma aberration correction^44,45^, chromatic aberration correction in refractive optics^46^, and multiwavelength achromatic focusing^15^. Given that the individual metasurfaces of these works can be manufactured at low cost by using our method, we believe this work can provide an experimental foundation for compound metalenses to fully compensate for the chromatic aberration.

In conclusion, we demonstrate a PPML, in which unit structures consist of NPCs to reduce fabrication costs. The NPC is composed of a UV-curable resin as a matrix that includes TiO_2_ NPs. The effective *n* of the NPC is increased by the TiO_2_ NPs, so the NPC can be used directly as a material for metalenses. The mold pattern can be replicated by the NPC due to its UV-curable matrix. Therefore, the PPML can be fabricated with one step of NIL without any secondary operations such as thin-film deposition and etching, which decrease productivity and substrate compatibility. The fabricated PPML achieves a focusing efficiency of 33% at the target wavelength of 532 nm, and the efficiency can be further increased by optimization of structure configuration. Focusing and imaging properties of the PPML are comparable to those of the diffraction-limited system. As a prototype of low-cost metalenses, the PPML will be a fundamental step toward commercialization of metalenses.

## Methods

### Fabrication of master mold

A 720-nm thick layer of hydrogenated amorphous silicon (a-Si:H) was deposited on a fused silica substrate by plasma-enhanced chemical vapor deposition. The electron beam resist (495 PMMA A2, Micro chem) was spin-coated on the sample at 2000 rpm, then baked at 180 °C for 5 min. A conductive polymer (Espacer 300Z, Showa denko) was spin-coated on the resist at 2000 rpm to prevent charge accumulation. The pattern of the PPML was drawn on the resist by EBL (ELS-7800, Elionix). The sample was immersed in DI water for 1 min to remove the conductive polymer, then immersed in developing solution (MIBK:IPA=1:3, Micro chem) for 12 min at 0 °C. A 30-nm thick chromium (Cr) layer is deposited on the sample by electron beam evaporation. A lift-off process was conducted in acetone to define a Cr mask on the sample. Then, the a-Si:H thin film was etched along the Cr mask using inductively-coupled plasma reactive ion etching. The master mold was completed after removal of a Cr mask residue using Cr etchant.

### Synthesis of NPC

The NPC was prepared by mixing two solutions. One is a mixture of a monomer (Dipentaerythritol penta-/hexa-acrylate, Sigma-aldrich), photo-initiator (1-Hydroxycyclohexyl phenyl ketone, Sigma-aldrich) and MIBK, and the other is TiO_2_ NP-dispersed MIBK (DT-TIOA-30MIBK(N30), Ditto technology). The mixing ratio of two solutions was controlled to achieve a weight ratio of 4 wt% for TiO_2_ NPs, 0.7 wt% for monomer and 0.3 wt% for photo-initiator in the NPC.

### Replication process of PPML

The master mold was coated by liquid-phase SAM to improve mold release in a solution mixture of a 1000:1 volume ratio of hexane and (heptadecafluoro-1,1,2,2-tetra-hydodecyl)trichlorosilane (H5060.1, JSI silicone) for 10 min. *h*-PDMS solution was prepared by mixing 3.4 g of vinylmethyl copolymers (VDT-731, Gelest), 18 μL of platinum-catalyst (SIP6831.2, Gelest), 0.1 g of modulator (2,4,6,8-Tetramethyl-2,4,6,8-tetravinylcyclotetrasiloxane, Sigma-aldrich), 2 g of toluene and 1 g of siloxane-based silane reducing agent (HMS-301, Gelest). The *h*-PDMS was spin-coated on the master mold at 3000 rpm for 50 s, then baked at 70 °C for 2 h. A degassed mixture of a 10:1 weight ratio of PDMS (Sylgard 184 A, Dow corning) and its curing agent (Sylgard 184 B, Dow corning) was poured on the *h*-PDMS layer, then baked at 100 °C for 2 h. The soft-mold was released from the master mold, then coated with vapor-phase SAM by a typical vaporizing process using (tridecafluoro-1,1,2,2-tetrahydrooctyl)trichlorosilane (SIT8174.0, Gelest) for 30 min at 5 Torr followed by DI water for 10 min at 10 Torr. The 3 μL of NPC was dropped on a glass substrate, then it was covered by the soft-mold. UV light was illuminated for 5 min at 2 bar to harden the NPC (Nanosis 820, NND). The soft-mold was released to complete the replication process.

## Supplementary information

Supplementary Information

## Data Availability

The data that support the findings of this study are available from the corresponding author upon reasonable request.
