# Supplementary material for: Single-step manufacturing of hierarchical dielectric metalens in the visible
Source: Nat Commun. 2020 May 8;11:2268. doi: 10.1038/s41467-020-16136-5 (PMC7211027; doi:10.1038/s41467-020-16136-5)
Supplement: Supplementary file 1 — Supplementary Information [file 41467_2020_16136_MOESM1_ESM.pdf]

Supplementary Information

Single-step manufacturing of hierarchical dielectric metalens in the visible

Gwanho Yoon,[1†] Kwan Kim,[2†] Daihong Huh,[2] Heon Lee[2]* and Junsuk Rho[1,3,4]*

[1]*Department of Mechanical Engineering, Pohang University of Science and Technology (POSTECH), Pohang 37673, Republic of Korea*

[2]*Department of Materials Science and Engineering, Korea University, Seoul 02841, Republic of Korea*

[3]*Department of Chemical Engineering, Pohang University of Science and Technology (POSTECH), Pohang 37673, Republic of Korea*

[4]*National Institute of Nanomaterials Technology (NINT), Pohang 37673, Republic of Korea*

*E-mail: *jsrho@postech.ac.kr* (J.R.); *heonlee@korea.ac.kr* (H.L.)

Supplementary Figure 1

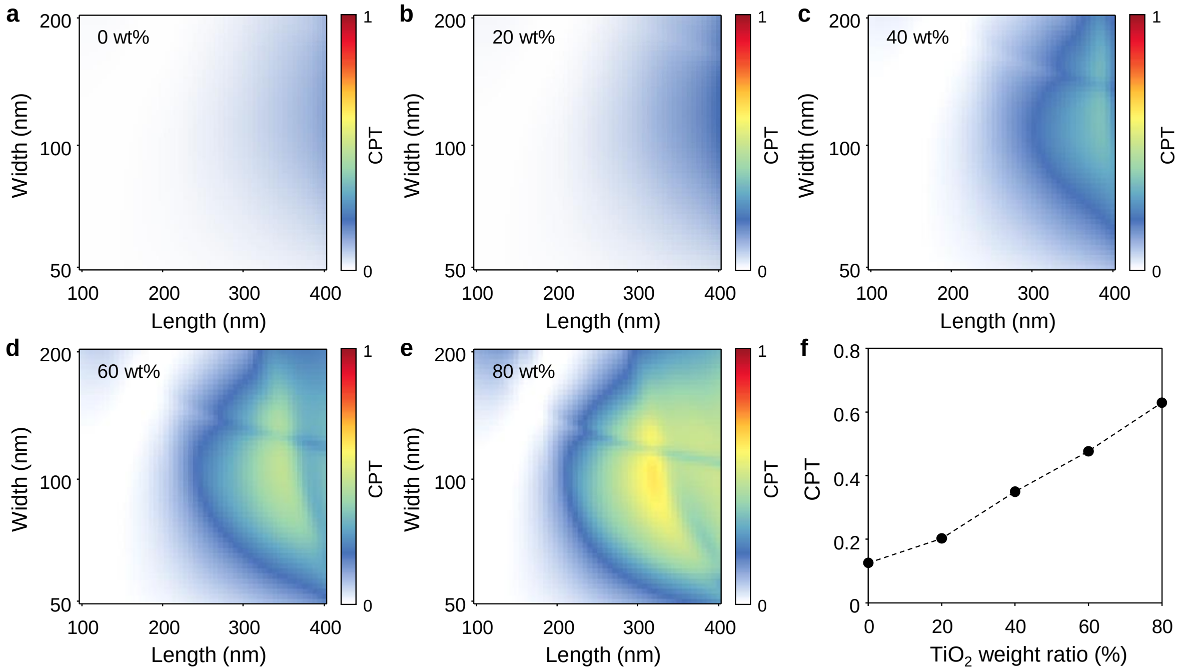

Figure 1. The effect of titanium dioxide (TiO_2) weight ratio on the metalens efficiency at the wavelength of 532 nm. **a-e** Calculated cross-polarization transmittance (CPT) of rectangular nanostructures using measured refractive indices of the nanoparticle composite. Height: 720 nm; pitch: 450 nm. The length varies from 100 nm to 400 nm while the width varies from 50 nm to 200 nm. **f** The maximum CPT variation according to the TiO_2 weight ratio. The dashed line is to guide the eye.

Supplementary Table 1

Table 1. Comparison of our method with typical nanoimprint lithography in the fabrication of dielectric metasurfaces

	Nanoimprint lithography (TiO_2 or GaN)	This work
**Thin-film deposition**	Necessary	Unnecessary
**Thin-film etching**	Necessary	Unnecessary
**Patterning on flexible substrate**	Not easy due to thermal damages from thin film deposition or etching steps	Possible
**Optical properties**	Controllable but very limited	Widely controllable by the content of nanoparticles
**General replication procedure**	1. Thin-film deposition on a substrate<br>2. Dropping the resist on the film<br>3. UV-NIL with a printing mold<br>4. Residue layer removal<br>5. Dry etching of the thin film<br>6. Resist residue removal	1. Dropping the NPC on a substrate<br>2. UV-NIL with a printing mold

TiO_2: titanium dioxide; GaN: gallium nitride; NPC: nanoparticle composite; NIL: nanoimprint lithography.

Supplementary Figure 2

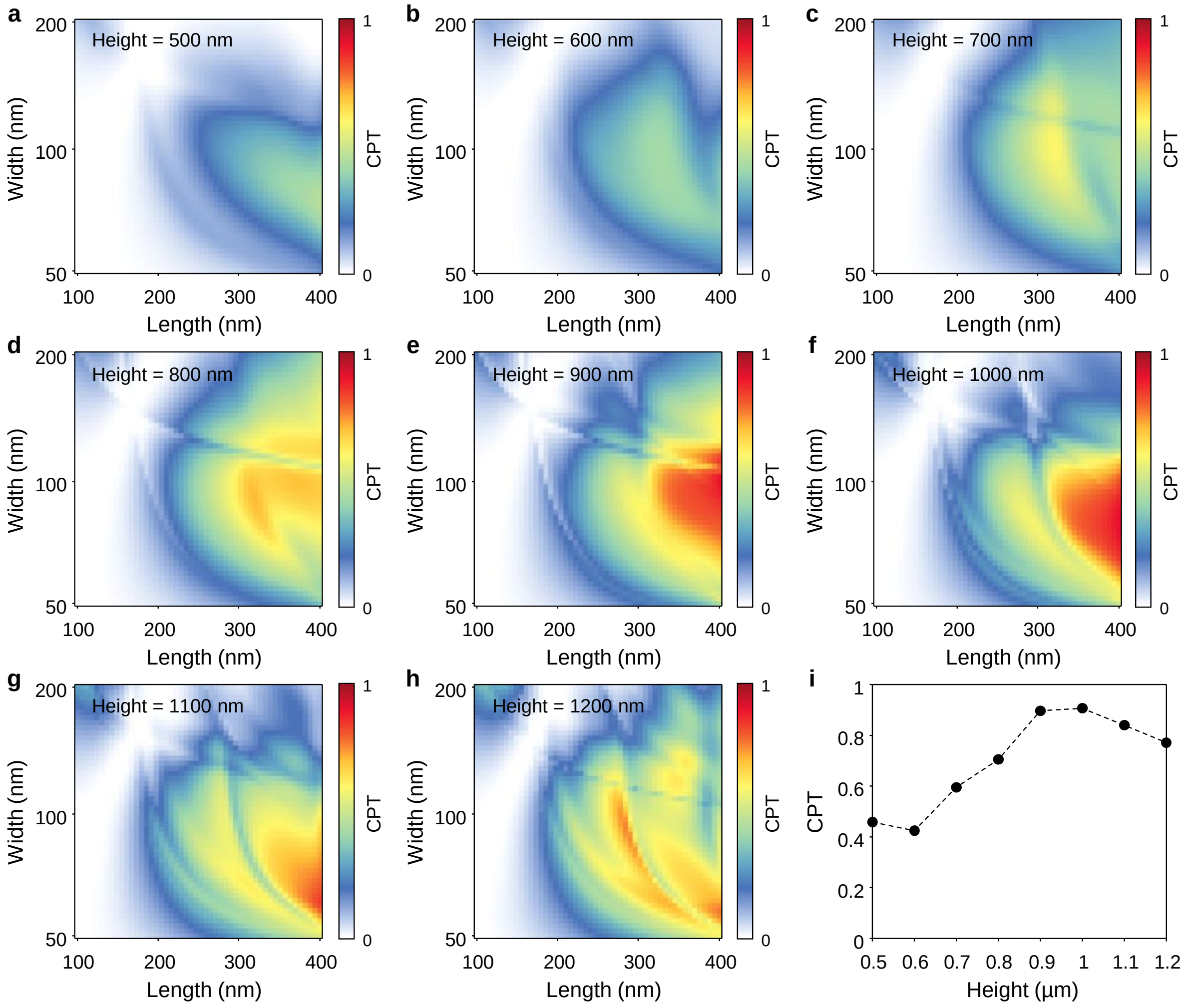

Figure 2. The effect of the structure height on the metalens efficiency at the wavelength of 532 nm. **a-h** Calculated cross-polarization transmittance (CPT) of rectangular nanostructures based on the nanoparticle composite. Pitch: 450 nm. The length varies from 100 nm to 400 nm while the width varies from 50 nm to 200 nm. **i** The maximum CPT variation according to the structure height. The dashed line is to guide the eye.
